# Functional discrimination of membrane proteins using machine learning techniques

**DOI:** 10.1186/1471-2105-9-135

**Published:** 2008-03-03

**Authors:** M Michael Gromiha, Yukimitsu Yabuki

**Affiliations:** 1 Computational Biology Research Center (CBRC), National Institute of Advanced Industrial Science and Technology (AIST), AIST Tokyo Waterfront Bio-IT Research Building, 2-42 Aomi, Koto-ku, Tokyo 135-0064, Japan

## Abstract

**Background:**

Discriminating membrane proteins based on their functions is an important task in genome annotation. In this work, we have analyzed the characteristic features of amino acid residues in membrane proteins that perform major functions, such as channels/pores, electrochemical potential-driven transporters and primary active transporters.

**Results:**

We observed that the residues Asp, Asn and Tyr are dominant in channels/pores whereas the composition of hydrophobic residues, Phe, Gly, Ile, Leu and Val is high in electrochemical potential-driven transporters. The composition of all the amino acids in primary active transporters lies in between other two classes of proteins. We have utilized different machine learning algorithms, such as, Bayes rule, Logistic function, Neural network, Support vector machine, Decision tree etc. for discriminating these classes of proteins. We observed that most of the algorithms have discriminated them with similar accuracy. The neural network method discriminated the channels/pores, electrochemical potential-driven transporters and active transporters with the 5-fold cross validation accuracy of 64% in a data set of 1718 membrane proteins. The application of amino acid occurrence improved the overall accuracy to 68%. In addition, we have discriminated transporters from other α-helical and β-barrel membrane proteins with the accuracy of 85% using k-nearest neighbor method. The classification of transporters and all other proteins (globular and membrane) showed the accuracy of 82%.

**Conclusion:**

The performance of discrimination with amino acid occurrence is better than that with amino acid composition. We suggest that this method could be effectively used to discriminate transporters from all other globular and membrane proteins, and classify them into channels/pores, electrochemical and active transporters.

## Background

Membrane proteins perform a diverse variety of functions, including the transport of ions and molecules across the membrane, bind to small molecules at the extra cellular space, recognize the immune system and energy transducers. The functional annotation of membrane proteins in genomic sequences is an important problem in bioinformatics and computational biology. Membrane transporters are a large group of proteins that span the cell membrane and form an intricate system of pumps and channels through which they deliver essential nutrients, eject waste products and assist the cell to sense environmental conditions. Transporters represent a large and diverse group of proteins that differ in membrane topology, energy coupling mechanism and substrate specificities [1]. They play indispensable roles in the fundamental cellular processes of all organisms [2].

Several methods have been proposed to discriminate membrane proteins from amino acid sequence information. These methods include statistical analysis [3-5], hidden Markov models [6,7] and machine learning techniques [8-10]. However, the discrimination of membrane proteins based on their functions is not yet explored and it is still at the infant stage.

In this work, we have analyzed the characteristic features of amino acid residues in major transporters, such as, channels/pores, electrochemical potential-driven transporters and primary active transporters. We have utilized different machine learning techniques for discriminating these classes of proteins and achieved the 5-fold cross-validation accuracy of 68%. The sensitivity of correctly identifying channels/pores, electrochemical and active transporters are, 55%, 70% and 76% respectively, in a set of 510, 502 and 706 proteins. The classification of channels and pores has been carried out, which showed the accuracy of 92%. In addition, we have discriminated transporters from other α-helical and β-barrel membrane proteins, and from all other proteins (globular and membrane) to the accuracy of 85% and 82%, respectively. Further, the influence of chain length for discrimination will be discussed.

## Methods

### Data sets

We have constructed datasets for channels/pores, electrochemical transporters and active transporters from the information available in Transport Classification Database, TCDB [11]. The TCDB has seven groups of transporters in which three of them have insufficient data for analysis and one is for incompletely characterized proteins. Hence, we have used the three major transporters, channels/pores, electrochemical and active transporters. The number of proteins belonging to these classes of transporters deposited in TCDB are 720, 989 and 1216, respectively. From these proteins, we have removed the redundant sequences using blastclust program [12] so that no two proteins have the sequence identity of more than 20%. This algorithm showed only one sequence in most of the clusters and we have randomly picked up one sequence for the clusters with many sequences. The final dataset contains 1718 proteins, which have 510 channels/pores, 502 electrochemical and 706 active transporters.

### Computation of amino acid composition and occurrence

The amino acid composition for the set of transporters has been computed using the number of amino acids of each type and the total number of residues. It is defined as:

(1) Comp(i) = Σ n_i_/N

where i stands for the 20 amino acid residues, n_i _is the number of residues of each type and N is the total number of residues. The summation is through all the residues in all the considered proteins. The same procedure was repeated for all the three groups of transporters for obtaining their amino acid composition. The total number of residues in the datasets of channels/pores, electrochemical and active transporters are respectively, 259,143, 252,585 and 289,109.

The amino acid occurrence is the actual number of amino acid residues of each type present in a protein without normalizing with chain length.

### 5-fold cross-validation method and jack-knife test

We have performed a 5-fold cross-validation test for assessing the validity of the present work. In this method, the data set is divided into five groups, four of them are used for training and the rest is used for testing the method. The same procedure is repeated for five times and the average is computed for obtaining the accuracy of the method.

In jack-knife test, n-1 data are used for training and the prediction is made on the left-out protein. This procedure is repeated for n times and the average is computed for obtaining the accuracy.

### Calculation of specificity, precision, F-measure and accuracy

We have used different measures, such as specificity, precision, F-measure and accuracy to assess the performance of discriminating channels/pores, electrochemical and active transporters. The term sensitivity shows the correct prediction of specific transporters and accuracy indicates the overall assessment. F-measure is the balance between sensitivity and precision, 1/F = [(1/Sensitivity) + (1/Precision)]/2. These terms are defined as follows:

Sensitivity = TP/(TP+FN)

Precision = TP/(TP+FP)

F-measure = (2 × Sensitivity × Precision)/(Sensitivity + Precision)

= 2TP/(2TP+FP+FN)

Accuracy = (TP+TN)/(TP+TN+FP+FN),

where, TP, FP, TN and FN refer to the number of true positives, false positives, true negatives and false negatives, respectively.

### Different machine learning algorithms used for discrimination

We have analyzed several machine learning techniques implemented in WEKA program [13] for discriminating membrane transporters from other proteins and classifying them into channels/pores, electrochemical and active transporters. This program includes several methods based on Bayes function, Neural network, Logistic function, Support vector machine, Regression analysis, Nearest neighbor, Meta learning, Decision tree and Rules. The details of all these methods are available in our earlier articles [9,10] as well as in the book on data mining [13].

## Results and Discussion

### Amino acid composition for the 20 amino acid residues in different transporters

The amino acid composition for the 20 amino acid residues in channels/pores, electrochemical and active transporters have been computed using Eqn. 1 and the results are presented in Table 1. Although several residues showed differences in their compositions, few residues have the difference of more than one (|Difference| >1) among the three classes of transporters. The residue Asn is dominant in channels/pores among all the transporters. Interestingly, Asn plays an important role to the stability and function of β-barrel membrane proteins [4,14]. The structural analysis on outer membrane cobalamin transporter protein (BtuB) that transports substrates across the outer membrane, showed that the residues, Asn185 and Asn276 are important for the stability of the upper surface of cyanocobalamin (vitamin B_12_; CN-Cbl) binding pocket [15,16], which is important for its function. In glycerol facilitator protein the residues Asn68 and Asn203 play important roles to the stability by making hydrogen bonds to form helical polar strips that connect the periplasmic and cytoplasmic versibules [17]. Glu is another amino acid that shows the difference of more than one with electrochemical transporters. It has been showed that the residues Glu166 and Glu148 are important for the channel function in CIC chloride channel proteins [18]. The composition of residues Ala, Ile and Leu in channels/pores are the least among the three transporters. Other hydrophobic residues also show similar tendency. It might be due to the fact that other two families are dominated with hydrophobic residues owing the presence of mainly transmembrane helical proteins.

**Table 1:** Amino acid composition in channels/pores, electrochemical and active transporters

Residue	Channels/pores	Electrochemical	Active
Ala	0.59	0.64	0.65
Asp	0.39	0.43	0.26
Cys	0.80	0.81	0.69
Glu	0.48	0.56	0.47
Phe	0.63	0.61	0.60
Gly	0.48	0.56	0.36
His	0.46	0.53	0.47
Ile	0.67	0.73	0.62
Lys	0.58	0.43	0.40
Leu	0.70	0.69	0.68
Met	0.63	0.63	0.49
Asn	0.41	0.36	0.38
Pro	0.26	0.44	0.28
Gln	0.49	0.51	0.56
Arg	0.52	0.60	0.47
Ser	0.49	0.44	0.43
Thr	0.59	0.62	0.48
Val	0.67	0.70	0.55
Trp	0.76	0.63	0.68
Tyr	0.71	0.67	0.54

The residues Phe and Leu are dominant in electrochemical transporters. In addition, the composition of Ala, Ile, Val and Trp are higher in this class of proteins compared with other two transporters. Interestingly, in glycerol-3-phosphate transporter the space between helices 1 and 7 is filled by nine aromatic side chains and the occurrence of bulky aromatic residues helps to close the pore completely [19]. In lactose permease the substrate binding site is composed of residues that include Trp151 [20]. The higher occurrence of hydrophobic residues is due to the presence of long stretches of these residues in membrane spanning segments of α-helical membrane proteins. The electrochemical transporters are mainly occupied with multiple spanning transmembrane helical proteins, which increased the occurrence of hydrophobic residues. On the other hand, the charged residues showed the lowest composition in this class of proteins. The composition of residues Asp, Glu and Lys are much lower than other transporters and Arg is also a less favored residue. However, the analysis of three dimensional structures showed that these charged residues are important for function. The residues Asp407, Asp480 and Lys940 are important for drug resistance in bacterial multidrug efflux transporter [21] and the charged residues E126, R144 and E269 are found to be in the substrate binding sites of lactose permease [20].

In active transporters none of the residue has the highest or lowest occurrence. All the residues have the composition, which lies between the compositions of channels/pores and electrochemical transporters. However, Glu, Gln, Phe, Arg and Lys are close to channels/pores whereas Ala, Asn, Thr and Tyr are close to electrochemical transporters. The structural analysis on high-potential iron-sulfur protein shows that the electron transfer is mainly achieved by hydrophobic interactions [22]. In addition aromatic residues are acting as binding site residues in vitamin B_12 _binding protein [23].

### Structural analysis of transporters

We have analyzed the three-dimensional structures of transporters deposited in TCDB and derived the propensity of the 20 amino acid residues to be in the membrane part. This has been computed by the ratio between the occurrence of each amino acid residue in the membrane part and the respective residue in the whole protein. The results obtained for channels/pores, electrochemical and active transporters are presented in Table 2. We observed that the membrane propensity of amino acid residues in channels/pores, electrochemical and active transporters have been partially reflected in their amino acid compositions. Especially the residues Asn and Tyr are dominant in channels/pores, the propensity of residues in active transporters is not the highest among all the three transporters, hydrophobic residues have high propensity in electrochemical transporters and so on. We noticed that the transporters will have 52–59% of their residues in the membrane part. It is noteworthy that the number of protein structures used to carry out the analysis is limited (a representative set of 22, 3 and 13 proteins in channels/pores, electrochemical and active transporters, respectively) and hence there may be a possibility of minor changes in results when more number of proteins are used in the analysis.

**Table 2:** Membrane propensity of amino acid residues in channels/pores, electrochemical and active transporters

Residue	Channels/pores	Electrochemical	Active
Ala	7.95	9.16	8.96
Asp	5.37	3.39	4.63
Cys	1.22	1.34	0.96
Glu	5.51	3.95	5.57
Phe	4.30	5.91	4.49
Gly	7.71	7.98	7.32
His	1.91	1.70	1.66
Ile	5.60	7.46	6.63
Lys	5.39	3.80	5.26
Leu	9.30	12.08	10.77
Met	2.21	2.99	2.74
Asn	5.34	3.39	4.04
Pro	4.13	4.39	4.48
Gln	4.03	2.89	3.81
Arg	4.74	3.85	4.66
Ser	7.64	7.36	6.47
Thr	5.94	5.60	5.62
Val	6.66	7.90	7.36
Trp	1.37	1.73	1.47
Tyr	3.58	3.13	3.00

### Performance of different machine learning techniques for discriminating channels/pores, electrochemical and active transporters

We have analyzed the performance of different machine learning methods for discriminating channels/pores, electrochemical and active transporters and the results obtained with amino acid composition are presented in Table 3. We observed that the sensitivity, precision and F-measure for electrochemical transporters is better than other two classes of proteins. The sensitivity, precision and F-measure for electrochemical transporters lies in the ranges of 0.58–0.82, 0.55–0.67 and 0.58–0.70, respectively. The values for channels/pores are 0.47–0.58, 0.53–0.68 and 0.51–0.59, and active transporters are 0.53–0.68, 0.55–0.62 and 0.56–0.65. The average accuracy of discriminating channels/pores, electrochemical and active transporters lies in the range of 56–64% for different machine learning techniques. The highest accuracy of 64% is obtained for neural network based method. Interestingly, this method has similar values with all measures indicating the ability of picking up the specific class of transporters and eliminating others with similar accuracy. In addition, we have tested the performance of the present method with jack-knife test and the results obtained with neural network are shown in Table 3. We noticed that the jack-knife test and 5-fold cross-validation showed similar results with a difference of 1.8%. We have also carried out the computations with same number of data in each class of transporters (502 proteins each) and we observed that the net accuracy (66%) is marginally better than that obtained with the original dataset.

**Table 3:** Discrimination of channels/pores, electrochemical potential-driven transporters and primary active transporters using different machine learning approaches with amino acid composition as features

Method	5-fold cross-validation									
	Sensitivity			Precision			F-Measure			Accuracy
	F1	F2	F3	F1	F2	F3	F1	F2	F3	(%)
Bayesnet	0.582	0.777	0.538	0.606	0.643	0.612	0.594	0.703	0.573	62.1
Naive Bayes	0.496	0.823	0.534	0.626	0.597	0.606	0.554	0.692	0.568	60.7
Logistic function	0.535	0.695	0.619	0.615	0.638	0.601	0.572	0.665	0.610	61.6
RBF network	0.543	0.735	0.625	0.640	0.666	0.603	0.587	0.699	0.614	63.3
Support vector machine	0.469	0.757	0.642	0.675	0.620	0.603	0.553	0.682	0.622	62.4
k-nearest neighbor	0.525	0.707	0.572	0.586	0.588	0.615	0.554	0.642	0.593	59.8
Bagging meta learning	0.541	0.679	0.677	0.646	0.660	0.618	0.589	0.669	0.646	63.6
Classification via Regression	0.492	0.695	0.630	0.599	0.628	0.599	0.540	0.660	0.614	60.8
Decision tree J4.8	0.506	0.580	0.572	0.529	0.581	0.554	0.517	0.580	0.563	55.5
NBTree	0.512	0.669	0.569	0.569	0.610	0.568	0.539	0.638	0.569	68.2
Partial decision tree	0.473	0.649	0.550	0.544	0.551	0.568	0.506	0.596	0.559	55.6
**Neural network**	**0.549**	**0.717**	**0.642**	**0.636**	**0.659**	**0.619**	**0.589**	**0.687**	**0.630**	**63.6**
Jack-knife test	0.571	0.709	0.676	0.664	0.660	0.644	0.571	0.709	0.676	65.4
Equal data	0.635	0.713	0.624	0.689	0.698	0.591	0.661	0.705	0.607	65.7

F1: channels/pores; F2: electrochemical potential-driven transporters; F3: primary active transporters. Equal data: Results obtained with a dataset of 502 proteins each in all the three classes of transporters.

Further, this analysis showed a moderate difference in the performance of different machine learning methods (the accuracy varies from 60% to 66% in most of the methods). The main cause of obtaining different prediction results might be due to the usage of different adjustable parameters in these methods.

### Influence of chain length for discrimination

The performance of different machine learning methods for discriminating channels/pores, electrochemical and active transporters with amino acid occurrence as features has been analyzed and the results are presented in Table 4. We observed that the average accuracy improved to 68% using neural network with amino acid occurrence. It has been shown that neural network is an efficient method for discriminating β-barrel membrane proteins [9,10]. The sensitivity is 0.55, 0.70 and 0.76 for channels/pores, electrochemical and active transporters, respectively. The precision is 0.70, 0.78 and 0.62, and F-measure is 0.61, 0.74 and 0.68. In addition, we have tested the performance of the present method with jack-knife test and the results obtained with neural network are shown in Table 4. We noticed that the jack-knife test and 5-fold cross-validation showed similar results with a difference of 2.7%. We have also carried out the computations with same number of data in each class of transporters (502 proteins each) and we observed that the net accuracy (68%) is similar to that obtained with the original dataset.

**Table 4:** Discrimination of channels/pores, electrochemical potential-driven transporters and primary active transporters using different machine learning approaches with amino acid occurrence as features

Method	5-fold cross-validation									
	Sensitivity			Precision			F-Measure			Accuracy
	F1	F2	F3	F1	F2	F3	F1	F2	F3	(%)
Bayesnet	0.329	0.735	0.567	0.554	0.515	0.572	0.413	0.606	0.569	54.6
Naive Bayes	0.202	0.757	0.575	0.477	0.512	0.534	0.284	0.611	0.554	51.8
Logistic function	0.533	0.713	0.705	0.689	0.717	0.604	0.601	0.715	0.651	65.
RBF network	0.247	0.727	0.633	0.486	0.593	0.530	0.328	0.654	0.577	54.6
Support vector machine	0.163	0.727	0.826	0.847	0.705	0.529	0.273	0.716	0.645	60.0
k-nearest neighbor	0.629	0.705	0.640	0.634	0.683	0.651	0.632	0.694	0.646	65.6
Bagging meta learning	0.553	0.685	0.737	0.676	0.733	0.625	0.608	0.709	0.676	66.7
Classification via Regression	0.465	0.721	0.721	0.686	0.702	0.602	0.547	0.711	0.656	64.5
Decision tree J4.8	0.543	0.625	0.555	0.526	0.592	0.593	0.534	0.609	0.574	57.2
NBTree	0.471	0.570	0.659	0.553	0.656	0.548	0.508	0.610	0.598	57.7
Partial decision tree	0.520	0.647	0.623	0.551	0.645	0.600	0.535	0.646	0.612	60.0
**Neural network**	**0.549**	**0.701**	**0.761**	**0.695**	**0.780**	**0.622**	**0.613**	**0.739**	**0.684**	**68.1**
Jack-knife test	0.500	0.703	0.729	0.639	0.749	0.607	0.561	0.726	0.663	65.4
Equal data	0.723	0.743	0.574	0.691	0.712	0.630	0.707	0.727	0.601	68.0

F1: channels/pores; F2: electrochemical potential-driven transporters; F3: primary active transporters. Equal data: Results obtained with a dataset of 502 proteins each in all the three classes of transporters.

The comparison of results presented in Tables 3 and 4 reveals that amino acid occurrence is better than composition for discriminating transporters. Recently, similar trend has been reported for discriminating different folding types of globular proteins [24]. These studies indicate the importance of chain length for discrimination in such a way that the normalization with chain length reduced the prediction accuracy.

When compared the performance of different machine learning methods, unlike amino acid composition, several methods showed poor sensitivity for channels/pores with occurrence. For example, Naïve Bayes showed the sensitivity of 0.20 and 0.76, respectively for channels/pores and electrochemical transporters. However, several methods (E.g. k-nearest neighbor, bagging, neural network etc.) showed good performances with similar sensitivity in all three classes of transporters.

### Comparison between the present method and the results obtained with BLAST search

We have analyzed the capability of BLAST to discriminate the three different types of transporters based on homology search. For each protein we have computed the sequence identity with all proteins in the three transporters and assigned the group, which has the highest sequence identity or best e-value. The calculations have been repeated for all the 1708 proteins and computed the overall accuracy. This method showed an accuracy of 51.6% in discriminating channels/pores, electrochemical and active transporters. Our method showed the accuracy of 75%, which is superior to simple BLAST search and the analysis revealed the better performance of the present method.

### Discrimination between two different classes of transporters

The amino acid composition of active transporters is in the range between that of channels/pores and active transporters (Table 1). Hence, we have examined the discrimination performance of two different transporters whether the discrimination accuracy is the highest between channels/pores and electrochemical transporters. The results are presented in Table 5. As expected the difference of amino acid compositions has been reflected in the performance of discrimination. The amino acid occurrence could discriminate the channels/pores and electrochemical transporters to the accuracy of 87%. The discrimination accuracy is 73% between channels/pores and active transporters, and 81% between electrochemical and active transporters. As discussed in previous sections, the discrimination accuracy with amino acid composition is less than that obtained with occurrence. However, we observed the same trend that the channels/pores and electrochemical transporters are discriminated with the highest accuracy.

**Table 5:** Discrimination accuracy between two different transporters

	5-fold cross-validation accuracy (%)		
	F1	F2	F3
**Occurrence**			
F1	-	86.8	73.2
F2	86.8	-	80.5
F3	73.2	80.5	-
**Composition**			
F1	-	81.4	71.8
F2	81.4	-	77.1
F3	71.8	77.1	-

Highest accuracy is shown.

F1: channels/pores

F2: electrochemical potential-driven transporters

F3: primary active transporters

### Discrimination of channels and pores

Proteins in the category of channels/pores have transmembrane channels, which consists of α-helical and β-strand spanning segments [11]. Hence, we have tested different machine learning algorithms to discriminate the channels (mainly α-helices) and pores (mainly β-strands). The results obtained with amino acid composition are shown in Table 6. We found that most of the machine learning methods discriminated the channels and pores with the accuracy in the range of 88–92%. The neural network and support vector machine showed the highest accuracy of 92.4%. The sensitivity and specificity are, 93% and 92%, respectively using neural network. We observed similar level of accuracy using amino acid occurrence. The classification via regression and logistic function methods discriminated the channels and pores with the accuracy of 90%. The similar performance with amino acid composition and occurrence might be due to the difference in amino acid residues in the membrane spanning regions of α-helical and β-barrel membrane proteins. The α-helical membrane proteins are dominated with the stretches of hydrophobic residues whereas the polar and charged resides are intervened in the membrane spanning segments of β-barrel membrane proteins. The high accuracy obtained for discriminating channels and pores is consistent with other methods for discriminating α-helical/β-barrel membrane proteins [3-10].

**Table 6:** Discrimination of channels and pores using different machine learning approaches

Method	5-fold cross-validation				
	Sensitivity (%)	Specificity (%)	F-measure		Accuracy (%)
			Channel	Pore	
Bayesnet	94.1	81.4	0.910	0.857	88.9
Naive Bayes	92.5	88.4	0.923	0.887	90.8
Logistic function	92.0	89.1	0.922	0.888	90.8
Neural network	93.0	91.5	0.935	0.915	92.4
RBF network	92.5	88.4	0.923	0.887	90.8
Support vector machines	95.2	88.4	0.937	0.905	92.4
k-nearest neighbor	89.8	86.8	0.903	0.862	88.6
Bagging meta learning	89.8	83.7	0.894	0.844	87.3
Classification via Regression	88.2	85.3	0.889	0.843	87.0
Decision tree J4.8	86.1	78.3	0.856	0.789	82.9
NBTree	90.9	83.7	0.899	0.850	88.0
Partial decision tree	87.2	79.1	0.865	0.800	83.9

### Discrimination of transporters from other membrane proteins and all other proteins

We have developed a dataset of 3336 membrane proteins with less than 20% sequence identity that includes receptors and all other types of α-helical and β-barrel membrane proteins except transporters from SWISS-PROT database. Using a dataset of 3336 non-transporters and 1718 transporters we have analyzed the performance of different machine learning algorithms and the k-nearest neighbor could discriminate the transporters with the 5-fold cross-validation accuracy of 79.1%. The sensitivity and specificity are 69.2% and 84.2%, respectively. Further, we have repeated the computations with equal number of transporters and non-transporters and obtained the accuracy of 85.0%. The jack-knife test also showed similar results that we obtained with 5-fold cross-validation method.

In addition, we have set up a dataset for 5048 proteins, which include membrane transport proteins and other membrane and globular proteins. We obtained a 5-fold cross-validation accuracy of 78.7% in discriminating transporters and non-transporters. Further, we have used the same number of proteins in transporters and non-transporters and repeated the calculations. We obtained the accuracy of 81.5% in distinguishing them, and both jack-knife test and 5-fold cross-validation method showed similar performance on discrimination.

### Discrimination on the web

We have developed web servers for (i) discriminating membrane transport proteins from all other membrane and globular proteins [25] and (ii) distinguishing channels/pores, electrochemical and active transporters [26]. These servers take the amino acid sequence as input and predict whether the protein is membrane transporter or not, and the type of the membrane transport protein. Both the servers can be freely accessible from our web site [27].

### Applications of the present method for new sequences

The following procedure may be used to detect the type of a new protein. First the new sequence can be identified as a transporter or non-transporter using the discrimination method to classify them (previous section). It has been shown that the transporters and non-transporters are discriminated with the highest accuracy of 82%. For a transporter, it can be further identified into channels/pores, electrochemical and active transporters with an accuracy of 68%. Alternatively, several methods have been reported in the literature for discriminating globular proteins from α-helical [3,28-30] or β-barrel [4-10,31] membrane proteins. These methods can be used to detect the membrane proteins. The membrane proteins of any kind can be classified into transporters and non-transporters with the maximum accuracy of 85%, and the transporters can be further classified into three groups. Hence, the two-way/three-way prediction system can be used to detect different types of transporters in genomic sequences. The work on the integration of prediction methods is on progress.

## Conclusion

We have systematically analyzed the amino acid compositions of channels/pores, electrochemical and active transporters and revealed the similarities and differences among them. Different machine learning algorithms have been tested to discriminate these transporters and we achieved the highest accuracy of 68% using neural network with amino acid occurrence. Further, we have examined the discrimination performance between two classes of transporters, which showed the highest accuracy of 87% between channels/pores and electrochemical transporters. In addition, the channels and porins are discriminated with the accuracy of 92%. On the other hand, the transporters and other membrane proteins/all other globular and membrane proteins are discriminated with the accuracy of 85% and 82%, respectively. We suggest that this method could be effectively used to discriminate transporters and different classes of transporters in genomic sequences.

## Availability and Requirements

Project name: Functional discrimination of membrane proteins

Project home page: 

Operating system(s): Platform independent

Programming language: Java

Licence: No restriction

Any restriction to use by non-academics: No restriction

## Authors' contributions

MMG conceived the project, carried out the computations and analysis, and wrote the manuscript. YY prepared the datasets and took part in computations. All authors read and approved the final manuscript.

## References

[B1] Ren Q, Chen K, Paulsen IT (2007). TransportDB: a comprehensive database resource for cytoplasmic membrane transport systems and outer membrane channels. Nucleic Acids Res.

[B2] Saier MH (2000). A functional-phylogenetic classification system for transmembrane solute transporters. Microbiol Mol Biol Rev.

[B3] Hirokawa T, Boon-Chieng S, Mitaku S (1998). SOSUI: classification and secondary structure prediction system for membrane proteins. Bioinformatics.

[B4] Gromiha MM, Suwa M (2005). A simple statistical method for discriminating outer membrane proteins with better accuracy. Bioinformatics.

[B5] Cai YD, Chou KC (2006). Predicting membrane protein type by functional domain composition and pseudo-amino acid composition. J Theor Biol.

[B6] Martelli PL, Fariselli P, Krogh A, Casadio R (2002). A sequence-profile-based HMM for predicting and discriminating beta barrel membrane proteins. Bioinformatics.

[B7] Bagos PG, Liakopoulos TD, Spyropoulos IC, Hamodrakas SJ (2004). A Hidden Markov Model method, capable of predicting and discriminating beta-barrel outer membrane proteins. BMC Bioinformatics.

[B8] Natt NK, Kaur H, Raghava GP (2004). Prediction of transmembrane regions of beta-barrel proteins using ANN- and SVM-based methods. Proteins.

[B9] Gromiha MM, Suwa M (2006). Discrimination of outer membrane proteins using machine learning algorithms. Proteins.

[B10] Gromiha MM, Suwa M (2006). Influence of amino acid properties for discriminating outer membrane proteins at better accuracy. Biochim Biophys Acta.

[B11] Saier MH, Tran CV, Barabote RD (2006). TCDB: the transporter classification database for membrane transport protein analyses and information. Nucleic Acids Res.

[B12] Altschul SF, Madden TL, Schaffer AA, Zhang J, Zhang Z, Miller W, Lipman DJ (1997). Gapped BLAST and PSI-BLAST: a new generation of protein database search programs. Nucleic Acids Res.

[B13] Witten IH, Frank E (2005). Data Mining: Practical machine learning tools and techniques.

[B14] Gromiha MM, Suwa M (2007). Current developments on β-barrel membrane proteins: sequence and structural analysis, discrimination and prediction. Curr Prot Pept Sci.

[B15] Chimento DP, Mohanty AK, Kadner RJ, Wiener MC (2003). Substrate-induced transmembrane signaling in the cobalamin transporter BtuB. Nat Struct Biol.

[B16] Chimento DP, Kadner RJ, Wiener MC (2003). The Escherichia coli outer membrane cobalamin transporter BtuB: structural analysis of calcium and substrate binding, and identification of orthologous transporters by sequence/structure conservation. J Mol Biol.

[B17] Fu D, Libson A, Miercke LJ, Weitzman C, Nollert P, Krucinski J, Stroud RM (2000). Structure of a glycerol-conducting channel and the basis for its selectivity. Science.

[B18] Dutzler R, Campbell EB, MacKinnon R (2003). Gating the selectivity filter in ClC chloride channels. Science.

[B19] Huang Y, Lemieux MJ, Song J, Auer M, Wang DN (2003). Structure and mechanism of the glycerol-3-phosphate transporter from Escherichia coli. Science.

[B20] Abramson J, Smirnova I, Kasho V, Verner G, Kaback HR, Iwata S (2003). Structure and mechanism of the lactose permease of Escherichia coli. Science.

[B21] Murakami S, Nakashima R, Yamashita E, Yamaguchi A (2002). Crystal structure of bacterial multidrug efflux transporter AcrB. Nature.

[B22] Nogi T, Fathir I, Kobayashi M, Nozawa T, Miki K (2000). Crystal structures of photosynthetic reaction center and high-potential iron-sulfur protein from Thermochromatium tepidum: thermostability and electron transfer. Proc Natl Acad Sci USA.

[B23] Borths EL, Locher KP, Lee AT, Rees DC (2002). The structure of Escherichia coli BtuF and binding to its cognate ATP binding cassette transporter. Proc Natl Acad Sci USA.

[B24] Taguchi YH, Gromiha MM (2007). Application of amino acid occurrence for discriminating different folding types of globular proteins. BMC Bioinformatics.

[B25] DISC-GLOB-MEMB-TRANSPORT. http://tmbeta-genome.cbrc.jp/disc-glob-memb-transport/.

[B26] DISC-TRANSPORT. http://tmbeta-genome.cbrc.jp/disc-transport/.

[B27] DISC-FUNCTION. http://tmbeta-genome.cbrc.jp/disc-function/.

[B28] Tusnady GE, Simon I (1998). Principles governing amino acid composition of integral membrane proteins: application to topology prediction. J Mol Biol.

[B29] Rost B, Casadio R, Fariselli P, Sander C (1995). Prediction of helical transmembrane segments at 95% accuracy. Protein Sci.

[B30] von Heijne G (1992). Membrane protein structure prediction. J Mol Biol.

[B31] Garrow AG, Agnew A, Westhead DR (2005). TMB-Hunt: a web server to screen sequence sets for transmembrane beta-barrel proteins. Nucleic Acids Res.

